# Impact of air temperature and drug concentration on liquid emission from elastomeric pumps

**DOI:** 10.1186/s40780-020-00185-5

**Published:** 2021-01-05

**Authors:** Tomoya Abe, Kazumasa Matsuzaka, Toshiaki Nakayama, Masanobu Otsuka, Atsunobu Sagara, Fumiaki Sato, Tetsuro Yumoto

**Affiliations:** 1 Department of Pharmacy, Saitama Cancer Center, 780 Komuro, Inamachi, Saitama, Japan; 2 Division of Pharmacy Professional Development and Research, Hoshi University School of Pharmacy and Pharmaceutical Sciences, 2-4-41 Ebara, Shinagawa-ku, Tokyo, Japan

**Keywords:** Elastomeric pumps, 5-fluorouracil, Air temperature, Emission rate, Chemotherapy, Viscosity

## Abstract

**Background:**

Elastomeric pumps (EPs) are devices that allow quantitative and continuous drug administration without the need for electronic control, and they are used by being filled with anticancer agents. Although the package inserts of several manufacturers that provide EPs describe the relationship between the flow rate per unit time and temperature, the solution is only saline solution or 5% glucose solution, and data on anticancer drugs have not been published. In this study, we focused on 5-fluorouracil (5-FU), a drug frequently used in cancer chemotherapy, and examined the effect of changes in standard of EPs and temperature on drug emission.

**Methods:**

We evaluated the EP data of patients treated with Baxter Infusor® LV5 and SV2.5 in terms of emission rate, relationship between 5-FU prescription amount and emission rate, and relationship between emission rate and monthly air temperature in LV5 and SV2.5. The number of EPs sampled in the study was *N* = 5708 (*n* = 2988 for LV5 and *n* = 2720 for SV2.5).

**Results:**

In LV5, the emission rate varied from 88 to 97% (median 94.0%), whereas in SV2.5, the emission rate was observed as 97 to 98% (median 97.4%). The 5-FU prescription amount and the emission rate were not correlated in LV5 and SV2.5, respectively (LV5; y = − 0.0015x + 97.305, *R*^2^ = 0.0226, SV2.5; y = − 0.001x + 100.25, *R*^2^ = 0.0466). LV5 showed a higher emission rate in the months with higher air temperature and a lower emission rate in the month with lower air temperature. In addition, LV5 showed a significant reduction in emission rate compared with SV2.5 in all months (*P* < 0.001).

**Conclusions:**

In this study, we clarified that air temperature is an important factor that affects the drug emission of EPs. Therefore, it is necessary to examine the conditions for total fluid volume suitable for the air temperature in each region and to provide sufficient information to patients.

## Background

Elastomeric pumps (EPs) are devices that allow quantitative and continuous drug administration without the need for electronic control, and are used by being filled with antibacterial agents, analgesics, and anticancer agents [1–4]. For cancer chemotherapy, the regimens FOLFOX and FOLFIRI for colorectal cancer and FOLFIRINOX for pancreatic cancer require continuous administration of 5-fluorouracil (5-FU) for 46 h [5–7]. Infusion pumps or EPs can be used for the continuous administration of these agents. In the clinical setting, it is not possible to completely eliminate human error that occurs as a result of an incorrect speed setting of the infusion pump, which can lead to a serious medical accident. On the other hand, EPs are highly safe medical devices that do not require a speed setting, and they discharge a drug solution at a constant speed. With recent advances in cancer chemotherapy, it has been reported that the shift from inpatient to outpatient treatment reduces the burden on patients and improves treatment satisfaction. EPs are useful even if the patient is treated at home instead of in the hospital, because they enable continuous drug administration [8, 9]. Therefore, EPs are used frequently.

Because EPs are produced by multiple medical device manufacturers, standards can vary, resulting in differences in the flow rate and filling amount per unit time depending on these standards. Furthermore, the positional relationship between the EP device and the discharge port, the viscosity of the drug, and temperature are assumed to be factors that affect the discharge of the drug from the EP.

Although the package inserts of several EP manufacturers describe the relationship between the flow rate per unit time and temperature, the solution used is only saline solution or 5% glucose solution, and data on anticancer drugs administered through EPs have not been published. In addition, the drug concentrations in EPs differ for each patient, and it is expected that the amount of drug discharge per unit time may differ depending on drug viscosity. Furthermore, in regions such as Japan where the temperature fluctuates by season, it is expected that the flow rate of the drug may also differ depending on the change in temperature. Against this background, there are no reports using actual drugs such as 5-FU, although various factors that influence the drug emission from the EPs are assumed in the clinical setting.

In this study, we focused on 5-FU as one of the drugs frequently used in cancer chemotherapy and examined the effect of variations in EP standards and changes in temperature on drug emission.

## Methods

From July 2008 to August 2013, we investigated the EPs of patients who received drug administration using the Baxter Infusor® (Baxter Healthcare Corporation, Pennsylvania, United States) at the Saitama Cancer Center. Our hospital uses LV5 and SV2.5 as the standard for the Baxter Infusor®. LV5 was prepared by mixing 5-FU (Kyowa Hakko Kirin Co., Ltd., Tokyo, Japan) and saline solution (FUSO Pharmaceutical Industries, Ltd., Osaka, Japan, or Hikari Pharmaceutical Co., Ltd., Tokyo, Japan) to achieve a total of 230 mL, and SV2.5 was prepared by mixing 5-FU and saline solution to achieve a total of 100 mL. Data were collected as follows: the date and time of preparation for each EP, the weight of the EP before drug filling, the weight of the EP after drug filling, the amount of the 5-FU prescription, and the weight of the EP 46 h after the start of drug administration. Based on these data, we examined the emission rate, the relationship between the 5-FU prescription amount and the emission rate, and the relationship between the emission rate and the monthly air temperature in LV5 and SV2.5. The air temperature was taken as the average temperature in 2013 in Saitama city, Japan [10]. The emission rate was calculated using the following method:

Emission rate (%) = EP weight after preparation − EP weight after administration × (5-FU liquid volume (mL)/(5-FU + saline solution volume [mL])/1.045 × 50)/5-FU prescription amount (mg) × 100 × 1.045: 5-FU specific gravity (g/mL) 50:5-FU concentration(mg/mL).The statistical difference between the LV5 and SV2.5 medians was determined using a Mann–Whitney *U* test. All statistical analyses were performed using IBM SPSS version 22. A *p* value < 0.05 denoted statistical significance.

## Results

**Fig. 1:**
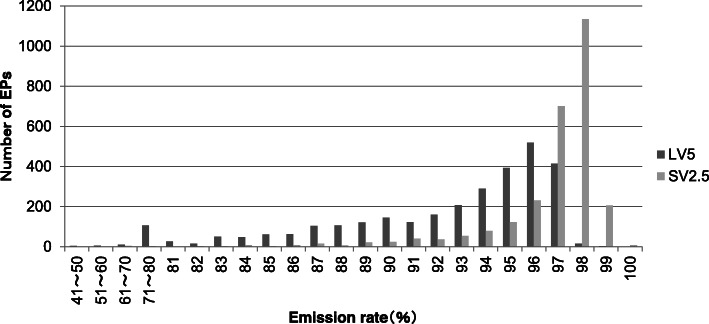
Emission rate distribution of LV5 and SV2.5 (LV5, *n* = 2988; SV2.5, *n* = 2720)

We first examined the difference in the 5-FU emission rate between LV5 and LV2.5 (LV5, *n* = 2988, SV2.5, *n* = 2720). Our results showed that in LV5, the emission rate of 5-FU varied from 88.0 to 97.0% (median 94.0%), whereas in SV2.5, a stable emission rate of 5-FU was observed from 97.0 to 98.0% (median 97.4%), (Fig. 1).

**Fig. 2:**
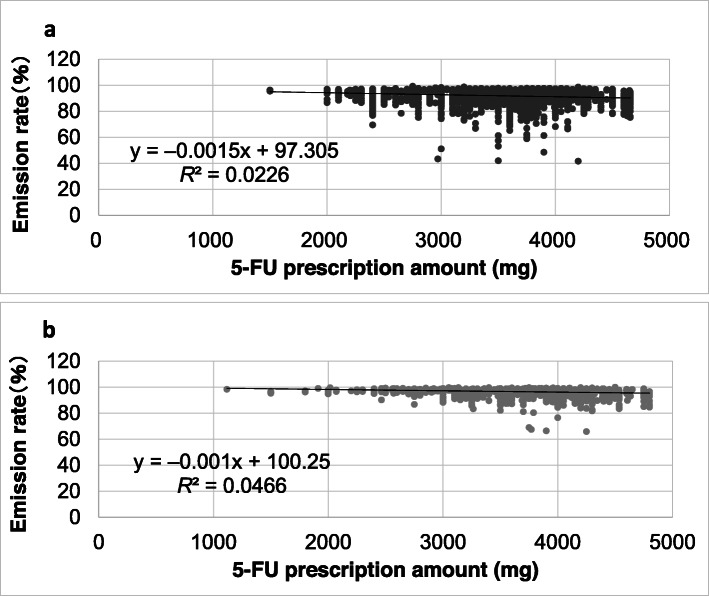
**a** Amount of 5-FU prescription and emission rate distribution in LV5. **b** Amount of 5-FU prescription and emission rate distribution in SV2.5

Next, we investigated the correlation between the amount of the 5-FU prescription and the emission rate in LV5 and SV2.5. We found no correlation between the 5-FU prescription amount and the emission rate in each group (Fig. 2; LV5 *y* = − 0.0015x + 97.305, *R*^2^ = 0.0226, SV2.5 *y* = − 0.001x + 100.25, *R*^2^ = 0.0466). We examined the relationship between the emission rate of 5-FU and the monthly average air temperature in Saitama city.

**Fig. 3:**
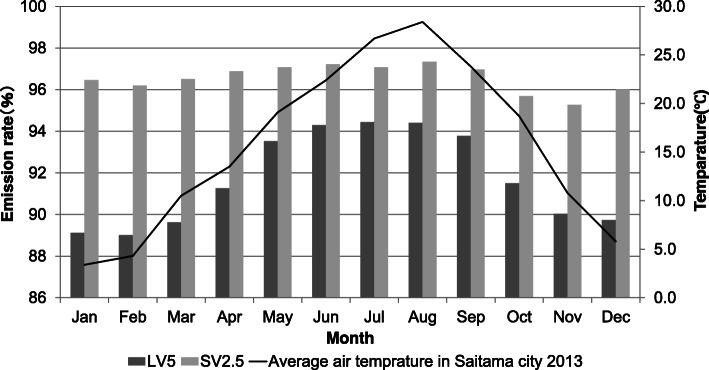
Relationship between the emission rate and the monthly air temperature in Saitama. For all months, LV5 showed a significant reduction in emission rate as compared with SV2.5 (*P* < 0.001) (January LV5 *n* = 220, SV2.5 *n* = 247; February LV5 *n* = 202, SV2.5 *n* = 220; March LV5 *n* = 208, SV2.5 *n* = 249; April LV5 *n* = 212, SV2.5 *n* = 249; May LV5 *n* = 202, SV2.5 *n* = 342; June LV5 *n* = 213, SV2.5 *n* = 321; July LV5 *n* = 350, SV2.5 *n* = 297; August LV5 *n* = 355, SV2.5 *n* = 246; September LV5 *n* = 331, SV2.5 *n* = 79; October LV5 *n* = 248, SV2.5 *n* = 134; November LV5 *n* = 242, SV2.5 *n* = 132; December LV5 *n* = 205, SV2.5 *n* = 204)

As compared with SV2.5, LV5 showed a higher emission rate in the months with higher air temperature and a lower emission rate in the month with lower air temperature. In addition, LV5 showed a significant reduction in emission rate as compared with SV2.5 in all months (Fig. 3; LV5 vs SV2.5, January 89.1% vs 96.2%, February 89.0% vs 96.2%, March 89.6% vs 96.5%, April 91.3% vs 96.9%, May 93.5% vs 97.1%, June 94.3% vs 97.2%, July 94.4% vs 97.1%, August 94.4% vs 97.3%, September 93.9% vs 97.0%, October 91.5% vs 95.7%, November 90.0% vs 95.3%, December 89.7% vs 96.0%, *P* < 0.001).

## Discussion

To provide appropriate cancer chemotherapy, it is important to confirm that the prescribed drug has been adequately administered. Bioavailability is generally used for oral drugs, which is a measure of how well the administered drug is absorbed into the body and exerts its effect. On the other hand, the bioavailability of drug administration by intravenous injection is 100%. However, in the case of administration using EPs, it is unavoidable that a certain amount of liquid remains inside the device after administration. Therefore, even if the prescription dose is estimated to be 100%, there is a difference in the dose into the body. The residual liquid volume after emission was about 3 mL the for LV5 and about 1 mL for SV2.5, which was about 1% of the total liquid volume, and it has been reported that the adverse effect is very small [11]. However, in this study, we confirmed that the emission rates were about 88 to 97% for LV5 and about 97 to 98% for SV2.5. From these results, we demonstrated that the residual liquid volume was about 3 to 12% for LV5 and about 2 to 3% for SV2.5, which is more than the manufacturer’s nominal 1%.

Next, we focused on 5-FU viscosity as a factor related to drug emission and examined the correlation between the amount of the 5-FU prescription and the emission rate. As shown in a previous report [8], we confirmed that there was no correlation between the amount of 5-FU prescribed and the emission rate (Fig. 2).

When we examined the emission rate and monthly air temperature, we found a higher emission rate for LV5 in the months with higher air temperature and a lower emission rate in the month with lower air temperature (Fig. 3). The emission rate of SV2.5 was less affected by air temperature as compared with LV5, suggesting that the greater the flow rate per unit time, the more likely it is to be affected.

Sato et al. reported no correlation between the residual fluid volume of 5-FU and air temperature, and a negative correlation between the administration time error and air temperature [12]. Other reports have recommend that the total liquid volume should be adjusted according to the EP standards and seasons [13, 14]. These reports agree with the results of the present study on the relationship between the prescription amount of 5-FU and emission rate, and the relationship between temperature and the emission rate. The strengths of this study compared with previous studies include a large sample size, longer study period, and use of multiple standard EPs. Therefore, our results provide more robust findings in comparison with these reports [13, 14] .

In addition to the viscosity of the drug and the temperature, which are factors that affect drug emission, there is a positional relationship between the EP device and the discharge port. In daily life, it is difficult to maintain the positional relationship between the EP device and the discharge port. Because there are sufficient cases in this study, the degree of influence on the positional relationship was expected to be small. Because the Baxter Infusor® is used by fixing the discharge port to the skin, we initially expected that there would be no significant difference in drug emission, because the skin temperature was kept constant. However, in contrast to expectations, we found that the effect of air temperature was greater than that of skin temperature, because there was a large difference in the monthly emission rate.

In the present study, factors other than temperature and viscosity that affect the emission rate were not investigated; therefore, there might be other factors that correlate with the emission rate, e.g.,. For example, venous pressure and the cover material used while carrying EPs. As per previous report, the venous pressure is very small compared with the internal pressure of the reservoir of the EP body [15]. The effect on the emission rate is considered limited; however, the effect may depend on the type and material of CV port and cover while carrying Eps. However, under clinical conditions, it became clear that the effect of temperature on the emission rate was significant. It is necessary to investigate these conditions to provide a more detailed correlation with emission rates.

The limitation of this study is the lack of information on therapeutic effects, such as prognosis and adverse events. If the administration time is significantly earlier or later than 46 h, the therapeutic impact remains unclear, such as whether all prescription drugs are therapeutically effective or prone to adverse events. In addition, it is unclear whether it is better to terminate the administration even if there is residual liquid 46 h after initiating the administration. Because these possibilities cannot be ruled out, the present study investigated the factors that affect the excretion rate to investigate optimal conditions for administering the prescribed dose at the optimal time.

When the continuous dose of 5-FU is 3200 mg/m^2^, as in FOLFOXIRI therapy [16], or when the patient’s body surface area is large, the volume of 5-FU will increase and EPs with a large standard may be used. In that case, the administration of a dose close to the prescribed amount is possible by adjusting the total liquid volume. Therefore, it is recommended to set the total liquid volume after carefully considering the local temperature and the diluted liquid volume. Manufacturers of EPs do not disclose the flow rate when filling with an anticancer drug such as 5-FU, and the total amount of liquid and the amount of diluted liquid are set at each medical facility. From the results of this study, we have revealed the emission rate of LV5 and SV2.5 of Baxter’s products, which has enabled us to provide useful information to facilities using the LV5 and SV2.5 EPs. We recommend that medical facilities in each region using not only the LV5 and SV2.5 but also various EPs examine the total liquid volume and promote information sharing among medical staff for medical safety management.

## Conclusion

In this study, we demonstrated that air temperature is an important factor that affects the drug emission of EPs. Therefore, the conditions for total fluid volume should be examined in terms of the suitability for the air temperature in each region and to provide sufficient information to patients.

## Data Availability

All the data generated or analyzed in this study are included in the published article.

## Abbreviations

EPs: Elastomeric pumps
5-FU: 5-Fluorouracil
